# Speaking up: using OSTEs to understand how medical students address professionalism lapses

**DOI:** 10.3402/meo.v21.32610

**Published:** 2016-11-03

**Authors:** Constance R. Tucker, Beth A. Choby, Andrew Moore, Robert Scott Parker, Benjamin R. Zambetti, Sarah Naids, Jillian Scott, Jennifer Loome, Sierra Gaffney

**Affiliations:** 1 Faculty Development, McGlothlin Medical Education Center, Virginia Commonwealth University School of Medicine, Richmond, VA, USA; 2 Department of Medical Education, University of Tennessee Health Science Center, Memphis, TN, USA; 3 Graduate Medical Education, University of Tennessee Health Science Center, Memphis, TN, USA

**Keywords:** professionalism, assessment, teaching and learning, medicine, simulation

## Abstract

**Background:**

Objective-structured teaching encounters (OSTEs) are used across many disciplines to assess teaching ability. The OSTE detailed in this paper assesses 191 fourth-year medical students’ (M4) ability to identify and address lapses in professionalism based on Association of American Medical Colleges’ professionalism competencies. The research questions addressed are

- How frequently do M4s address professionalism lapses observed during an OSTE?
- What factors influence whether M4s provide feedback when they observe professionalism lapses in an OSTE?

**Methods:**

Standardized patients (SPs) and standardized learners (SLs) were recruited and trained to participate in a standardized encounter with specific cognitive, social, and behavioral errors, including professionalism lapses. M4s viewed this encounter and then offered feedback to the SL, while remotely observed by faculty. Post-encounter, the SL and faculty completed identical checklists to assess both teaching readiness and ability to address professionalism concerns.

**Results:**

An analysis of frequencies showed that six of the Association of American Medical Colleges’ nine professional competencies were addressed in the checklist and/or discussed in the focus group. Analysis of transcribed debriefing sessions confirmed that M4s did not consistently address professionalism lapses by their peers.

**Conclusions:**

In focus groups, M4s indicated that, while they noticed professionalism issues, they were uncomfortable discussing them with the SLs. Findings of the current study suggest how medical educators might support learners’ ability to address lapses in professionalism as well as topics for future research.

Teaching and evaluating professionalism in undergraduate medical education is the focus of much research and discussion in recent years (1–5). Lapses in professional behaviors around the world are prompting initiatives to strengthen professionalism training. However, the very nature of professionalism is contested within the literature as either a concept or a set of behaviors, skills, values, or attitudes (6, 7). The foundation of medical professionalism is defined by Swick (4, p. 613) as ‘the values and behaviors that individual physicians demonstrate in their daily interactions with patients and their families, and with physicians and other professional colleagues’. He argues that these behaviors must show that physicians and, by extension, medical students are worthy of their patients’ trust. In the 2005 *Recommendations for Clinical Skills Curricula for Undergraduate Medical Education* published by the Association of American Medical Colleges (AAMC), professionalism is defined as ‘the ability to understand the nature of, and demonstrate professional and ethical behavior in, the act of medical care. This includes the competencies of respect, responsibility and accountability, excellence and scholarship, honor and integrity, altruism, leadership, cultural competency, caring and compassion, and confidentiality’ (8). For the purpose of this study, professionalism is defined according to these competency-based criteria.

Papadakis et al.'s (1) retrospective review of the link between professionalism lapses in medical school and later disciplinary action by state medical boards emphasizes why professionalism must be addressed during medical education: 95% of these board disciplinary actions were for deficiencies in professionalism. Study physicians disciplined by the medical board of California were twice as likely as physicians in the control group to have negative evaluative narratives in their medical school file. In response to studies like these, professionalism is taught, assessed, and evaluated in various ways including checklists, narrative writing, mentorship, observation, and recognition efforts (9–11). Despite the numerous methods, all aim to increase learner's reflective ability. Hoffman et al. (12) examined the relationships of reflective ability and professionalism lapses during medical school and suggested that activities that engage student reflection can promote professional behavior. Although researchers cannot reach consensus on the most appropriate method, no study to date has examined the use of simulation to teach, assess, or evaluate medical student professionalism (13). This study examines how and why medical students identify and address professionalism lapses during an objective-structured teaching encounter (OSTE).

OSTEs are used across many disciplines to assess teaching ability. Teaching ability, in this study, is defined as the ability to observe clinical encounters and offer feedback. A typical OSTE set-up involves a standardized patient (SP), a standardized learner (SL), the teacher who is being evaluated, and a faculty observer (14–16). The teacher observes the SL treat the SP and then offers direct oral feedback to the SL as to how to improve patient care (4). After this exchange, both SL and faculty observer complete checklists that evaluate specific teaching components.

OSTEs are a valuable tool because they are well received by participants and allow interested parties to view and evaluate teachers in a realistic environment (14). In most medical OSTEs, third- or fourth-year medical students are the typical SLs as the teaching evaluation is focused on residents or attending physicians. Current literature does not consider use of the OSTE for assessing medical students as teachers (17). This study takes the novel approach of assessing fourth-year medical students (M4s) through an OSTE format where recruited participants played the role of SLs while M4s were evaluated on their ability to teach and provide feedback. The OSTE additionally permitted M4s to consider their future role as residents, where it is assumed that teaching skills are developed, although these skills are often not explicitly taught in a structured framework in many institutions.

This OSTE permits observation of medical student responses to a simulated encounter where an SL portrays an undergraduate medical student demonstrating multiple (scripted) behavioral, social, and cognitive lapses (18). In this standardized environment, the fourth student, soon to become a resident as teacher, is able to observe and provide feedback to the SL, but also be observed and receive feedback from medical faculty and SLs.

The primary goal of this research is to examine how and when M4s address professionalism lapses that are deliberately scripted into an OSTE. Research questions explored in the study are:

- How frequently do M4s address professionalism lapses observed during an OSTE?
- What determines whether M4s provide direct feedback if they observe professionalism lapses in an OSTE?

## Methods

This research was reviewed and approved by the human subjects protection committee of the University of Tennessee Health Science Center Institutional Review Board.

### Participants

A single-station OSTE was administered at the University of Tennessee Health Science Center (UTHSC) College of Medicine in April 2014 and 2015 through the Principles of Clinical Medicine course. Participation was voluntary, and M4s were able to choose from multiple sessions to allow for scheduling flexibility. Over 2 years, 191 M4s participated. No students received prior formal preparatory training on teaching; all had participated in at least one professionalism presentation at orientation.

### Procedure

SPs and SLs were recruited, trained, and compensated for their time. Four UTHSC employees were compensated in the form of their hourly wage, while hired actors were paid $15 per hour. Faculty observers were invited to participate on a voluntary basis.

**Fig. 1:**
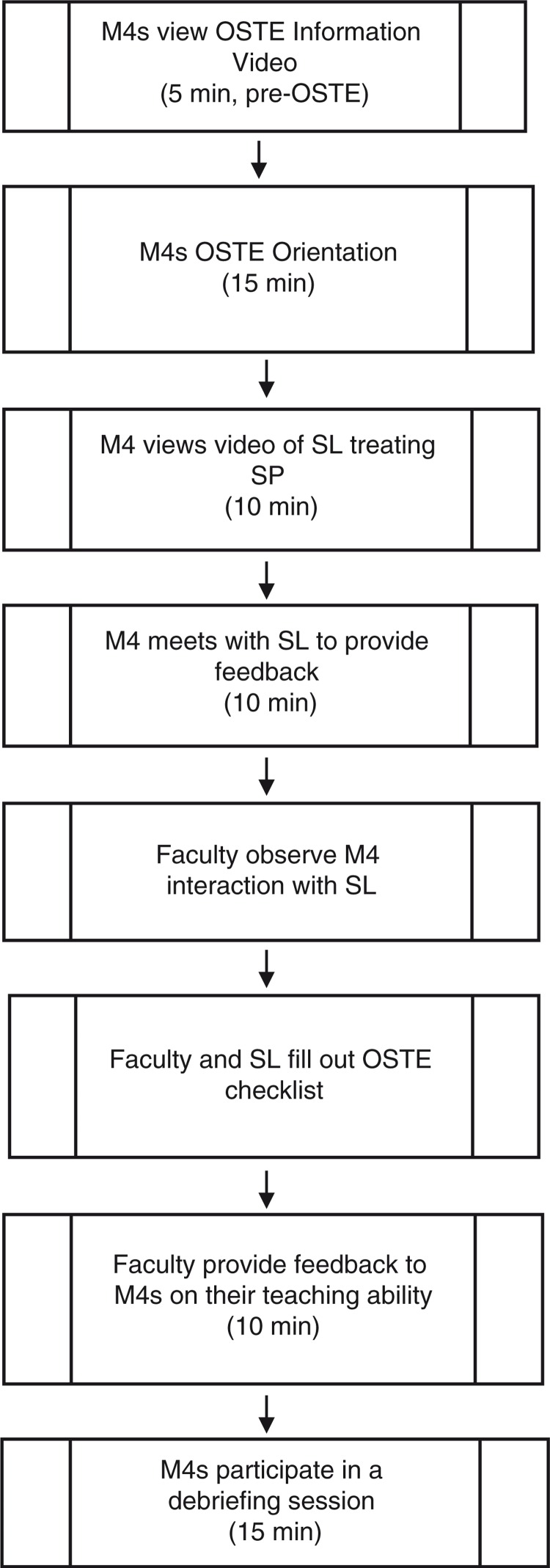
The OSTE procedure.

Figure 1 describes the entire OSTE process used in this study. M4s read Ende's article on feedback in the clinical learning environment and watched a 3-min ‘Orientation to the OSTE’ video prior to their OSTE appointment (19). In addition, M4s attended a 15-min OSTE orientation on the day of their experience in which researchers provided an overview of the study (19). Both the online and live orientation explained the purpose of engaging in the OSTE and introduced a framework for teaching and providing feedback in the clinical setting. Each M4 then watched a prerecorded video of an SL interviewing and examining the SP. All M4s watched an identical video. After watching the video, M4s were asked to write down the three most important issues to address with the student when providing feedback. These three items were drawn from the M4s’ personal assessments of the video; no checklists or other assessments were used at this time. After the M4s completed their note sheets, they read the following note ‘You have just watched a video of an M1 student's history and physical exam. Please provide the M1 with feedback on the encounter’. The M4 then entered the exam room to provide feedback to the SL using that written sheet. Faculty observed real-time recordings of this session. Post-encounter, both SL and faculty completed checklists to assess the M4's ability to observe clinical encounters and offer feedback The M4s then participated in a 10-min feedback session with the faculty observer and SL. Finally, M4s participated in a 15-min guided debriefing session to discuss their OSTE experience with other M4s and a trained facilitator (Appendix 1). All debriefing sessions were audio recorded and later transcribed.

### OSTE case design

The OSTE case design was developed to assess the ability of M4s to observe clinical encounters and offer feedback that addressed lapses in professionalism. The scenario was designed to demonstrate typical mistakes made by first-year medical students during a 10-min targeted exam. A common cold scenario was chosen to maintain the focus on providing feedback to the SL rather than developing the differential diagnosis and treatment plan. Professionalism related clinical errors were scripted into the scenario through the use of compound questions, disorganized/incomplete history and physical, inappropriate draping, missed hand hygiene, inappropriate social history interviewing style, lack of attention to patient comfort, poor interpersonal communication, overuse of medical terminology, and unprofessional attire.

To determine if the M4s addressed professionalism lapses by the SL, checklists were developed using *Recommendations for Clinical Skills Curricula for Undergraduate Medical Education, Association of American Medical Colleges, 2008, Appendix 1: Professionalism* list of competency goals. Checklists were developed using four predefined steps, including drafting a preliminary checklist and having content experts review this draft and then edit and resubmit the final version to experts for secondary review (20). At completed development, six of the nine categories from the AAMC list were included in the checklist: Respect, Excellence and Scholarship, Honor and Integrity, Cultural Competency, and Caring, Compassion, and Confidentiality.

### Data collection

#### Quantitative

**Fig. 2:**
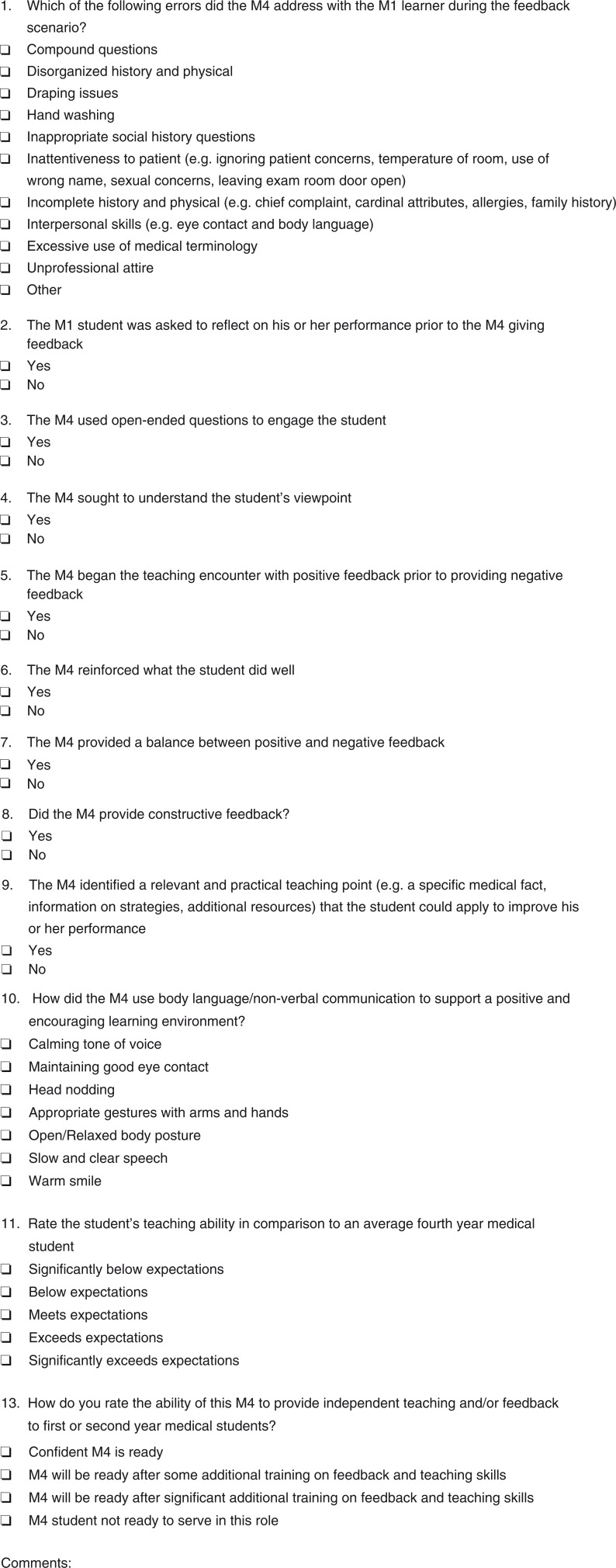
OSTE checklist for faculty observers.

Faculty observer and SL checklists were collected for each student participating in the OSTE during both 2014 and 2015. Student data were excluded if checklists were missing (i.e., the faculty observer missed the first part of the encounter). In 2014, 89 faculty and SL checklists were collected. In 2015, 101 faculty and SL checklists were collected. The frequencies with which the fourth-year students addressed the SL's professionalism lapses were calculated using IBM's Statistical Package for the Social Sciences 22 SPSS. The SLs and faculty groups were compared using independent paired *t*-tests, 95% CI, with *p*<0.05 significant, to determine if the two raters (SL or faculty) reported different frequencies. The faculty observer and the SL completed identical checklists for each M4 either while or immediately after the SL feedback by the M4 (Fig. 2).

#### Qualitative

All data from the debriefing sessions were digitally recorded and subsequently transcribed. Sixteen sessions were recorded and transcribed in 2014 and 23 sessions were recorded and transcribed in 2015. Transcripts were analyzed using three coding levels, including initial coding, constant comparison, and theoretical coding (21). Initial coding included the identification and frequency of key words. Constant comparison occurred within individual researcher analysis. Inter-rated reliability was also evaluated using comparisons of selected transcripts between all researchers. Cronbach's alpha value for 2014 was 0.830; it was 0.970 for 2015. The third level of coding included theoretical coding in which the AAMC professionalism competency goals were used to develop a theoretical understanding of the data. Throughout the analytical process, researchers created memos to explain their coding process and identify significance and notable relationships within the data.

To strengthen the qualitative results, triangulation of data was used to facilitate a deeper understanding (22). First, methodological triangulation was achieved by integrating both qualitative and quantitative data into the study. Second, triangulation of sources was achieved by comparing students with different viewpoints at unique points in time over 2 years. Thirdly, analyst triangulation was achieved by using multiple analysts to review findings as well as the use of multiple observers during the OSTE activities.

## Results

**Fig. 3:**
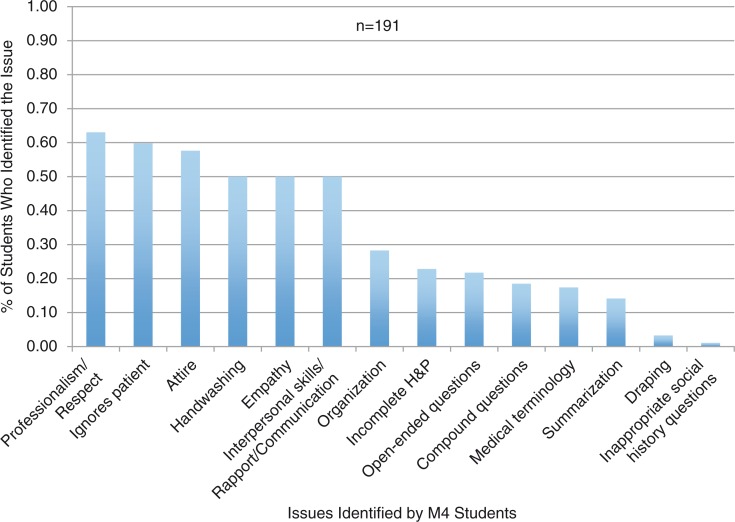
Professionalism issues identified on M4 note sheet.

To answer the question as to how frequently M4s address professionalism lapses observed during an OSTE, the frequency of deliberate professionalism lapses discussed by the M4s with the SLs was examined (Table 1). To answer the second research question, the researchers transcribed and coded post-OSTE debriefing sessions to determine why M4s either addressed or avoided professionalism lapses. To best align with the AAMC professional competencies, the quantitative and qualitative findings were reported under each AAMC construct for both years (Fig. 3).

**Table 1:** AAMC professionalism competency goals and the corresponding checklist items

AAMC professionalism competency goal	Checklist item
Respect	Lack of hand washing
	Unprofessional attire
Excellence and scholarship	Recognize and manage: Disorganized history and physical
	Incomplete history and physical
Honor and integrity	Introduction of M4 to the patient
Cultural competency	Use of compound questions
	Excessive use of medical terminology Admitting mistakes and errors
Caring and compassion	Inappropriate social history questions
	Inattentiveness to patient
	Poor interpersonal skills
Confidentiality	Closing exam room door/respecting privacy Appropriate draping

AAMC, Association of American Medical Colleges; M4, fourth-year medical student.

### Issues noted by M4 students on the M1 video

The M4 note sheets provided insight as to what the medical students saw as professionalism lapses prior to providing oral feedback to the SL. Summative results of the transcribed data from these note sheets are presented in Fig. 3. The most common issues identified by students were grouped under professionalism/respect, ignoring the patient, and attire. The least common issues identified on student note sheets were inappropriate social history questions and inconsideration of patient modesty (ineffective draping of the SP during exam). Examples of comments on students note sheets are provided in the next section.

**Professionalism/Respect**

MS 40: It's okay to go out of order, but make sure to keep patient informed. Respecting the patient is most important.

MS 3: She did not speak to the patients in a way that he would feel comfortable and understand what was going on.

**Ignoring the Patient**

MS 89: Ignores patient concerns – i.e., sexual problems.

MS 43: Listen to patient when you ask them a question.

MS 62: Did not address patient concerns (decreased sex drive).

**Attire**

MS 39: Attire…

MS 27: Professional dress. Short skirt.

MS 46: Dress – button shirt.

**Draping**

MS 3: She did not drape the patient appropriately during the exam, leaving him exposed the entire time

MS 76: Draping! – Minimal exposure, don't leave undraped.

MS 33: Never leave patient undraped.

**Inappropriate social history questions**

MS 41: Weird social history – you're married?

MS 5: Presumptive social history, rude

MS 91: Awkward social history – made assumptions

The M4s wrote numerous statements documenting that they were aware of professionalism lapses after watching the video. Some students simply listed the errors in professionalism, while others wrote directly to the SL and provided corrective statements.

### Observations of professionalism during OSTE

Faculty and SL used a checklist to assess when they observed the M4s addressing unprofessional behavior. After verifying inter-rater reliability using chi-squared goodness of fit test, results were organized in aggregate. The faculty and SL observed 191 students during the OSTE. The competencies observed in these M4 students included honor and integrity 189 (99.2%), respect 101 (52.6%), caring and compassion 99 (51.8%), and cultural competency 53 (28.4%). Confidentiality could not be reliably assessed on the faculty and SL checklists, and was subsequently removed.

### Competency goals and M4 debrief

During the M4 debrief, students responded to the guided facilitator questions with rich qualitative narrative (Appendix 1). The narratives were focused primarily on two AAMC competencies: caring/compassion and respect. Of the 171 coded responses over 2 years, 136 codes addressed competencies of caring and compassion (66) and respect (70). While medical students discussed the other competencies, the coded response rate was much lower. The remaining competencies were coded a total of 35 times, including excellence and scholarship (22), honor and integrity (5), cultural competency (4), and confidentiality (4).

### Caring and compassion

Topics related to the competency of caring and compassion were highlighted as important in both the OSTE and the debriefing sessions. While many M4s represented that these competencies were important and should be addressed, they were much less comfortable discussing the SL's lack of interpersonal skills with the SL personally.

MS14: I picked a couple things that really focus on building rapport with the patient, and history gathering.

MS15: I felt like I was a little hesitant about bringing up the guys general slacker attitude, so I just tried to bring up some points that he could hopefully connect the dots to, like empathy, and patient comfort – how they are feeling in the room at that moment – sometimes you can't say to someone that you're kind of sloppy and slouchy.

MS17: I wanted to tell my guy that he had no people skills and couldn't relate to the patient even though the patient was obviously in distress, there was a disconnect. But I didn't know if that was just his personality, or if he was in a bad mood, so I didn't push the issue so much.

MS18: It did make a difference when talking with the patient, how you come off to them, and he was very coarse. I wanted to talk about that, but I didn't want to hurt his feelings because that is who he was. (MS Debrief)

According to student debrief narratives, personal relationship with peers and the desire to avoid conflict hindered direct and specific feedback about professionalism lapses in the competency of caring and compassion.

### Honor, integrity, and respect

The competencies of honor and integrity focused on the priorities of making the patient comfortable and introducing oneself. MS4s documented their observations of honor and integrity in their notes before the OSTE encounter and also during the debrief.

MS 12: I also prioritized patient safety, introducing yourself, saying you are a medical student.

MS13: Making the patient more comfortable: introduce yourself, shake hands, make eye contact. (MS Debrief)

Respect, the second of the two competencies, was most commonly discussed in the debrief sessions and was often related to the discussion of unprofessional attire. Students commented on their significant discomfort addressing the SL's attire (i.e., casual T-shirt and miniskirt). In contrast, students expressed no problem addressing other behaviors related to respect for the patient (i.e., draping), which made this competency relatively highly addressed.

MS4: I just thought about picking general themes. Hand washing, you say the patient's name wrong and you blame it on the nurse, just kind of saying, you know, to help build rapport. (MS Debrief)

MS17: [I avoided talking about] the dress. But as a male evaluating a female I'm not saying a thing about that. In the real world. I'm not saying anything about it. I've seen worse by MD attendings. What if my definition of conservative varies…it's too subjective.

A possible explanation for greater comfort addressing draping and hand washing as opposed to attire may stem from the types of errors that these different lapses represent. While draping and hand washing were viewed as more procedural, some M4s expressed that addressing their learner's personal ‘style of dress’ made them uncomfortable, as if they were attacking them personally.

MS3: It was the first thing you see. Number one on my list. It was glaringly obvious. Just point out how you should dress.

MS2: You have to be professional in the academic setting and then you go to the real world where no one is half as professional as they should be. So it felt weird to critique someone on professionalism. (MS Debrief)

MS91: Today you just can't do a whole lot of that without overstepping sexual harassment issues.

MS 115: I definitely called mine out on it. You can't wear a T-shirt and you need to wear a white coat. But your pants, socks, and shoes are all very appropriate.

Many students in the current study demonstrated discomfort addressing unprofessional attire. Reasons for the discomfort included opposite gender from the SL, unprofessional dress of attendings, and fear of retaliation.

### Excellence and scholarship

Learners seemed most comfortable bringing up lapses in the history and physical exam with their standardized ‘peers’. This could possibly be due to the less personal nature of the history taking process as opposed to discussing attire or interpersonal/communication skills. Students were also motivated to discuss history and physical as important to successful completion of the upcoming USMLE Step 2 Clinical Skills exam.

MS5: I picked a couple things that really focus on building rapport with the patient, and history gathering.

MS6: The first thing you have to do is be able to talk to somebody and get a history.

MS7: So the way I narrowed it down was everything that has to do with history taking and basically interaction with the patient, taking care of needs of the patient, whether they are cold or things like that are more important because that makes the patient more comfortable, you can get more information from them.

MS8: For instance, mine didn't do very good ROS, his physical exam was lacking, and the open-ended questions as well, but I also tried to hit on the points that probably get docked the most on the CS exam. (MS Debrief)

In 2015, this competency was discussed in the transcripts as being easy to address, but did not translate into a high mean on the faculty/SL checklists. One explanation for this contrast may be the result of what some students discussed as grouping. The students grouped several professionalism lapses into an overarching theme for their learner (i.e., poor interpersonal skills was grouped with clear communication).

### Cultural competency

Cultural competency relates to the ability to effectively communicate with and gather information from diverse patient populations (i.e., age, primary spoken and/or written language, race/ethnicity, disability, religion, gender, sexual orientation, health literacy level and socioeconomic status) (23). Issues related to cultural competency were one of the least often addressed topics discussed by the M4s. In the transcripts, students generally commented on communication but did not discuss specific errors (i.e., using medical jargon, speaking too fast) made by the learner.

MS9: Communication, etiquette that was sort of evolving too. That'll come with time, and I think it's important to point that out, too.

MS10: I tried to describe a way to communicate with the patient

MS11: I went about it by theme, like I went patient communication, physical exam, and developing a better history, and so addressing each one of those themes. (MS Debrief)

The low frequency of addressing this specific competency and the low frequency of mention during the debriefing session may suggest that students did not find this competency as important as others or that students did not perceive a relationship between communication and cultural competency within patient encounters.

### Confidentiality

While the competency of confidentiality was not reliably captured on the faculty and SL checklists, it was captured in the medical student debriefs. Students consistently mentioned the importance of draping and privacy.

MS19: You know what you should do with draping, respect the privacy, you could address communication and protecting privacy and it will go a long way.

MS20: I tried to lump mine into three categories: patient-physician relationship, physical exam, and patient privacy.

MS21: I mean the major things were like respecting patient privacy or bodily integrity, keeping covered. I feel like that was really stressed with us, and I think it's important that it is.

The note sheets and debrief session provided insight into the students’ recognition of draping and patient privacy. Students demonstrated that this competency was emphasized within their curriculum and reiterated in their training.

### Debrief

During the debrief, when asked what students took away from the OSTE experience, students spoke to their ability to provide feedback, utilize questioning, and stay organized.

MS25: The feedback, like I kinda just started listing things that went wrong. I didn't start out with something positive. They mentioned you need to start out with something positive with the kid because they are nervous. They said to me a lot of people will start focusing on every little thing or don't hear half of what you are saying.

MS26: I liked something my M1 [SL] said to me actually. He said to make it more engaging. I was going to bring up draping. He said one way you can bring up draping is starting off with a question. How did you feel about draping? If they say ‘I nailed it’ then you know to be a little more sensitive. If they were uncomfortable then you can be like, that's good because it was uncomfortable to watch.

MS27: Trying to learn how to be more organized as a teacher. I've never done it before so I felt a lot of what I was saying I just didn't have a good organizational structure for communicating the top three.

MS28: I think we all have had experiences during third and fourth year where we interact with residents and attendings and think to ourselves, I hope I never act this way with medical students or people who are under me. And I think that this type of situation is good in that it gives us an opportunity to criticize people in a setting where you can assess what you are doing.

MS30: It's a weird line you have to draw. You have to be professional in the academic setting and then you go to the real world where no one is half as professional as they should be. So it felt weird to critique someone on professionalism. My M1 wasn't wearing his white coat and I need to tell him he needs to wear his white coat all the time but there have been several times where I have been interviewing real live patients and not been wearing my white coat or have been wearing tennis shoes or there might be a blood stain because I just got off of surgery. Real life is real life. It's kind of weird to preach at people when you know that's not always the case.

Medical students described a great deal of satisfaction with the OSTE experience. However, there was a tension between what they were taught in the OSTE and what occurs in the clinical teaching environment or ‘real life’.

## Discussion

The main purpose of this study was to determine how and why medical students address obvious professionalism lapses during an OSTE. A secondary objective of this study was to examine the reliability and validity of the OSTE assessment checklist specifically designed.

### Not so hidden curriculum

In the current study, students mentioned that the behaviors being reinforced in the OSTE were not always evident in clinical training. Students described professionalism as part of a hidden curriculum in which behaviors are reinforced and stabilized rather than taught (24, 25). The medical students were more likely to be influenced by the hidden curriculum than the OSTE training session. Students noted that the dark behaviors of social influencers (i.e., peers, physicians, nurses, and educators) contradict the professional ethics they learn during the preclinical years. Students reflected that all medical educators need to move the hidden curriculum from the dark into the light (26).

### Debriefing is powerful

Faculty and SL observed medical students engaging in all five professionalism competency goals. M4s addressed certain competency goals with differing frequencies, however. Medical students were most likely to address the competencies of honor and integrity, excellence and scholarship, and caring and compassion in the OSTE, but were less likely to bring up the competencies of respect and cultural competency. However, in the reflective debriefing session students’ the primary competency goals discussed were respect and compassion. Behavioral assessments like an OSTE with a reflective debriefing session may provide unique opportunities for students to demonstrate different aspects of professionalism. The OSTE provided an opportunity for students to demonstrate competency through excellence and scholarship, while the debriefing session provided a powerful narrative that illuminated competencies that otherwise would have been missed.

### Not all competencies are created equal

Not only are the definitions of professionalism varied and complex, professional behaviors are often interrelated, context-dependent, and in conflict with one another (27). When students describe professional behaviors in the study, they discussed the overlap and interdependence of attributes such as honor, integrity, and respect. Students spoke of honor, integrity, and respect and also highlighted how context changes professional behavior (i.e., male MS4s commented that they were uncomfortable discussing unprofessional attire with a female SL). The MS4 students also discussed having to choose some competencies over others. Some students discussed professionalism concerns with the SL when they were perceived to directly impact patient care; they were more avoidant if the professionalism competency was perceived as indirect to patient care. Some M4s wanted to address the lack of care and compassion for the SL, but decided that respect for their peers was more appropriate when the competencies seemed to conflict. Students acknowledged the complexity of these interrelated, context-dependent, and, at times, conflicting competencies and the impact they have on professional clinical behavior.

### Limitations and future directions

Some methodologic limitations affect this study. The OSTE as a competency-based teaching and assessment tool promoted professionalism as an opportunity for information collection rather than an opportunity to promote patient-centeredness seen with reflective narrative writing (10, 28). The current study was able to assess six of the nine AAMC competency goals. The three competency goals that were not assessed included responsibility and accountability, altruism, and leadership. Due to the inability to assess these three competency goals, the OSTE scripts and checklists reinforce traditional knowledge transmission from teacher to student rather than patient-centered engagement.

The competency checklists also lacked validity due to confounding behaviors. The checklist categorized hand washing with respect, although it could easily be associated with excellence and responsibility. Admitting mistakes or errors was categorized within cultural competency, but could also be associated with honor and integrity or responsibility. Professionalism is not only challenging to describe across cultures, individuals, and times, but also difficult to assess. While not wholly generalizable, findings are probably not unique to the sampled group, with some attitudes and viewpoints likely being similar to other learners at this stage of training.

Despite these limitations, this study demonstrated novel use of 191 M4s in an OSTE. As the majority of OSTE research to date has had sample sizes in the low 30s, this larger group provides information not before considered. Future studies of medical student responses to professionalism lapses using OSTE would benefit from continued work towards a reliable and valid checklist based on the AAMC's professional competency goals. A pre- and post-test OSTE to assess the effectiveness of a professionalism identity development course for medical students is another interesting topic for further exploration.

## Conclusion

The use of the OSTE to teach and assess professionalism is a unique way to train future medical educators to observe and address lapses. Professional behavior must be addressed early in medical education so professional identity/responsibility begins to develop. Proper acculturation into the medical profession is essential, with hopes that learners grow more comfortable addressing professionalism issues with their peers with time. While learners addressed professionalism lapses with their simulated peers, only five of nine AAMC competencies defined were addressed. The ability to provide peer feedback on issues of professionalism is important to maintain our profession. Self-awareness and self-regulation are integral features of professional behavior. If students are unaware of or fail to address lapses with peers while in medical school, will they do so after graduation?

Fourth-year medical students may feel neither comfortable nor responsible for addressing peer lapses. When students were asked about barriers to addressing professionalism, some highlighted a desire to avoid personal conflict in established peer relationships. Others mentioned that all professional competency goals are not considered as equal. Students also highlighted inconsistencies between preclinical and clinical experiences in which the importance of professionalism is not overtly modeled. The hidden curriculum in the third and fourth years may subtly undo earlier (preclinical) teaching regarding patient-centered communication and care. These challenges provide medical educators an opportunity to learn how to best engage colleagues in difficult conversations. Medical educators can use this information as they adapt curriculum to better students’ ability to engage these professionalism competencies. Through this research, students may begin to reflect, assess, and regulate their ability to identify address and model professional attitudes and behaviors.

**Practice Points**

- Observed simulated teaching encounters (OSTEs) are an important and effective process for faculty and students to practice, assess, and reflect on how to address professionalism lapses and what hidden curriculum exists that promotes or hinders professionalism.
- Post-OSTE student debriefs provided unique opportunities for students to engage in narrative storytelling and patient-centered reflections that a behavioral OSTE alone could not otherwise demonstrate.

## Appendix 1

Medical student debrief focus group guide

Introductory Protocol

To facilitate our note-taking, we would like to audiotape our conversations today. For your information, only researchers on the project will be privy to the tapes that will be eventually destroyed after they are transcribed.

We have planned this interview to last no longer than 20 min. During this time, we have several questions that we would like to cover. If time begins to run short, it may be necessary to interrupt you in order to push ahead and complete this line of questioning.

Students can share whatever aspects of their experiences that they feel comfortable revealing.

1. What was it like to receive immediate feedback from faculty members and from standardized learner?
2. What were the differences in the feedback you received from the faculty member and from the standardized learner?
3. How did you decide which mistakes to address?
4. Were there any issues you were not comfortable addressing that you completely avoided? If so, what were they?
5. How was the OSTE a beneficial exercise for improving teaching abilities?
6. What parts of the OSTE would you like to see improved?
  - If you viewed the OSTE training video, what improvements would you make to enhance your learning?
  - How realistic were the scenarios?
7. What will/would/could you do differently after having participated?
